# Revealing chemical processes and kinetics of drug action within single living cells via plasmonic Raman probes

**DOI:** 10.1038/s41598-017-02510-9

**Published:** 2017-05-23

**Authors:** Shan-Shan Li, Qi-Yuan Guan, Gang Meng, Xiao-Feng Chang, Ji-Wu Wei, Peng Wang, Bin Kang, Jing-Juan Xu, Hong-Yuan Chen

**Affiliations:** 1 State Key Laboratory of Analytical Chemistry for Life Science and Collaborative Innovation Center of Chemistry for Life Sciences, School of Chemistry and Chemical Engineering, Nanjing University, Nanjing, 210023 China; 2 Jiangsu Key Laboratory of Molecular Medicine, Medical School and the State Key Laboratory of Pharmaceutical Biotechnology, Nanjing University, Nanjing, 210093 China; 3 National Laboratory of Solid State Microstructures, College of Engineering and Applied Sciences and Collaborative Innovation Center of Advanced Microstructures, Nanjing University, Nanjing, 210093 China

## Abstract

Better understanding the drug action within cells may extend our knowledge on drug action mechanisms and promote new drugs discovery. Herein, we studied the processes of drug induced chemical changes on proteins and nucleic acids in human breast adenocarcinoma (MCF-7) cells via time-resolved plasmonic-enhanced Raman spectroscopy (PERS) in combination with principal component analysis (PCA). Using three popular chemotherapy drugs (fluorouracil, cisplatin and camptothecin) as models, chemical changes during drug action process were clearly discriminated. Reaction kinetics related to protein denaturation, conformational modification, DNA damage and their associated biomolecular events were calculated. Through rate constants and reaction delay times, the different action modes of these drugs could be distinguished. These results may provide vital insights into understanding the chemical reactions associated with drug-cell interactions.

## Introduction

Revealing molecular mechanisms of anticancer drugs is one of the most important tasks for drug research^1–3^. Precisely understanding the chemical processes and kinetics of drug action inside cells would definitely benefit drug mechanism research and new drug development^2, 4–6^. Although current molecular biological methodologies have displayed a magnificent picture about drug action targets and pathways, information related to their chemical processes and kinetics is still poorly understood thus far. From the view point of chemistry, cell is a complex, dynamic and heterogeneous system undergoing continuous matter transportation, energy exchange and chemical reactions^7^. Drug action on intracellular biomolecules (e.g. protein, DNA, RNA etc.) involve multiple chemical reactions, including bond breakage, conformational modification, native structures degradation and so on^8–10^. Finding out how these chemical reactions occurred, and understanding their reaction kinetics would provide extremely valuable information about drug interaction with cells. However, fully revealing the occurrence and kinetics of such processes are challenging, and demand an operando technique, which enables to simultaneously observe multiple biomolecular events and also give adequate chemical information to discriminate the difference.

Raman spectroscopy can readily provide abundant fingerprint information of various molecules in biological system and has shown promise in studying biomolecular events within living cells^11–16^. Particularly, plasmonic-enhanced Raman spectroscopy (PERS), using localized fields of plasmonic nanoprobes to enhance the Raman signals of molecules where they target, enables to monitor chemical changes at specific location of cells in real-time^14, 17–24^. If combining with statistical method, like principal component analysis (PCA) or linear discriminant analysis (LDA), the most principal information could be extracted from multidimensional spectral data, and different molecular communities could be discriminated^18, 21, 22^.

Herein, we attempted to make a step towards revealing the chemical kinetics of drug action at single cell level, using gold nanostars (AuNSs) with very strong localized plasmonic field to enhance the Raman signals from the region of cell nucleus. Choosing three types of commonly-used “nucleus-target” chemotherapy drugs (5-FU, CisPt and CAMP) as models, we studied the processes of drug induced chemical changes on proteins and nucleic acids via time-resolved PERS. The communities of chemical changes during the treatment processes of different anticancer drugs could be clearly discriminated through running PCA analysis on the PERS spectra. By fitting the dynamic changes of Raman bands with kinetic model, the rate constants and reaction delay times of protein denaturation, conformational modification, DNA damage and their associated biomolecular events were calculated, by which the difference on action modes of these three types of drugs could be distinguished.

## Results and Discussion

### Plasmonic probes and real-time PERS measurement of drug action process

**Figure 1:**
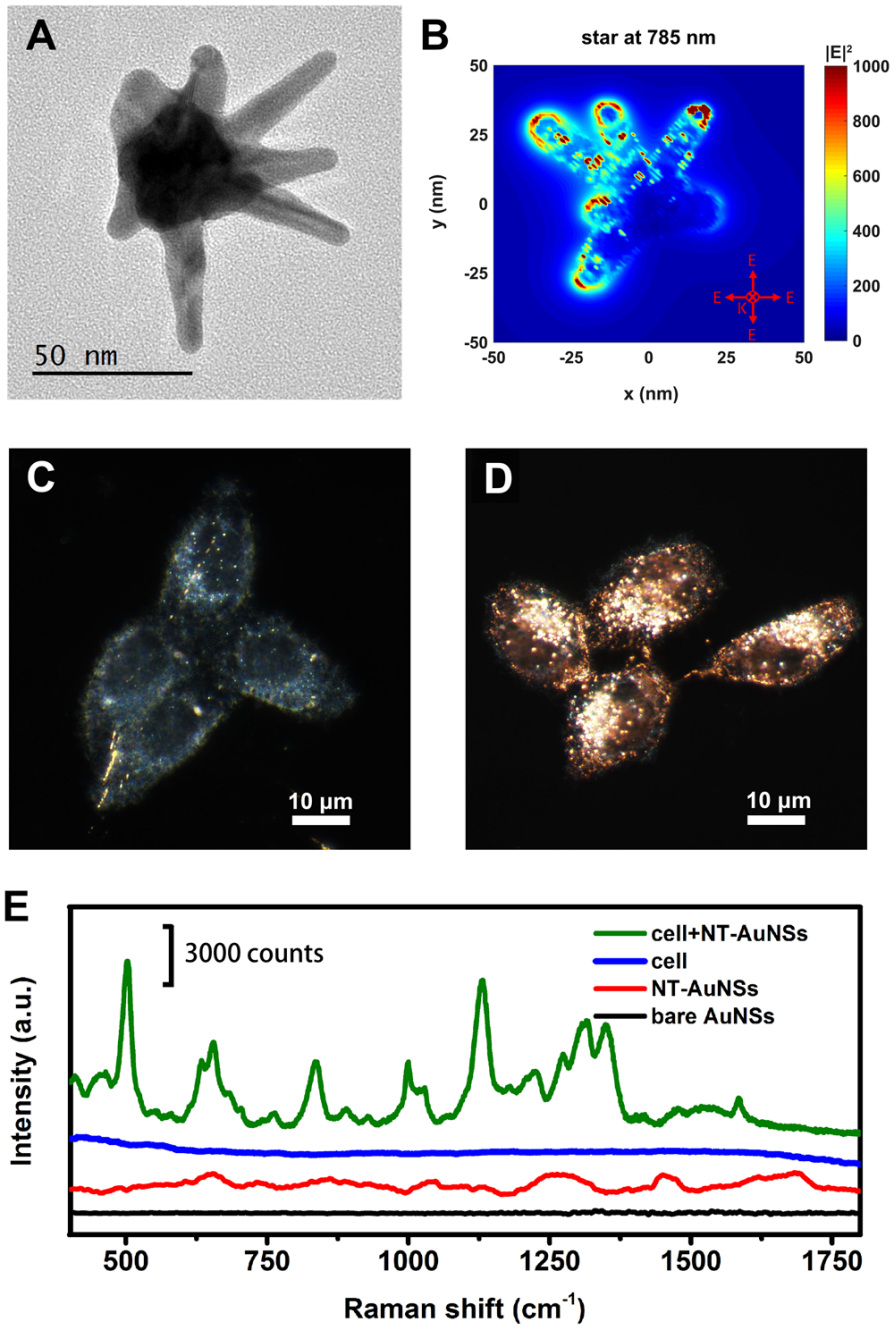
(**A**) Transmission electron microscopic (TEM) micrograph of AuNS. (**B**) Finite-Difference Time-Domain (FDTD) simulated distribution of localized electronic field of AuNS at Raman incident wavelength. Dark field images of MCF-7 cells pre-incubated without (**C**) and with (**D**) 0.05 nM NT-AuNSs for 24 h. (**E**) Typical Raman spectra of cells with and without NT-AuNSs, and the spectra of NT-AuNSs, and bare AuNSs.

**Figure 2:**
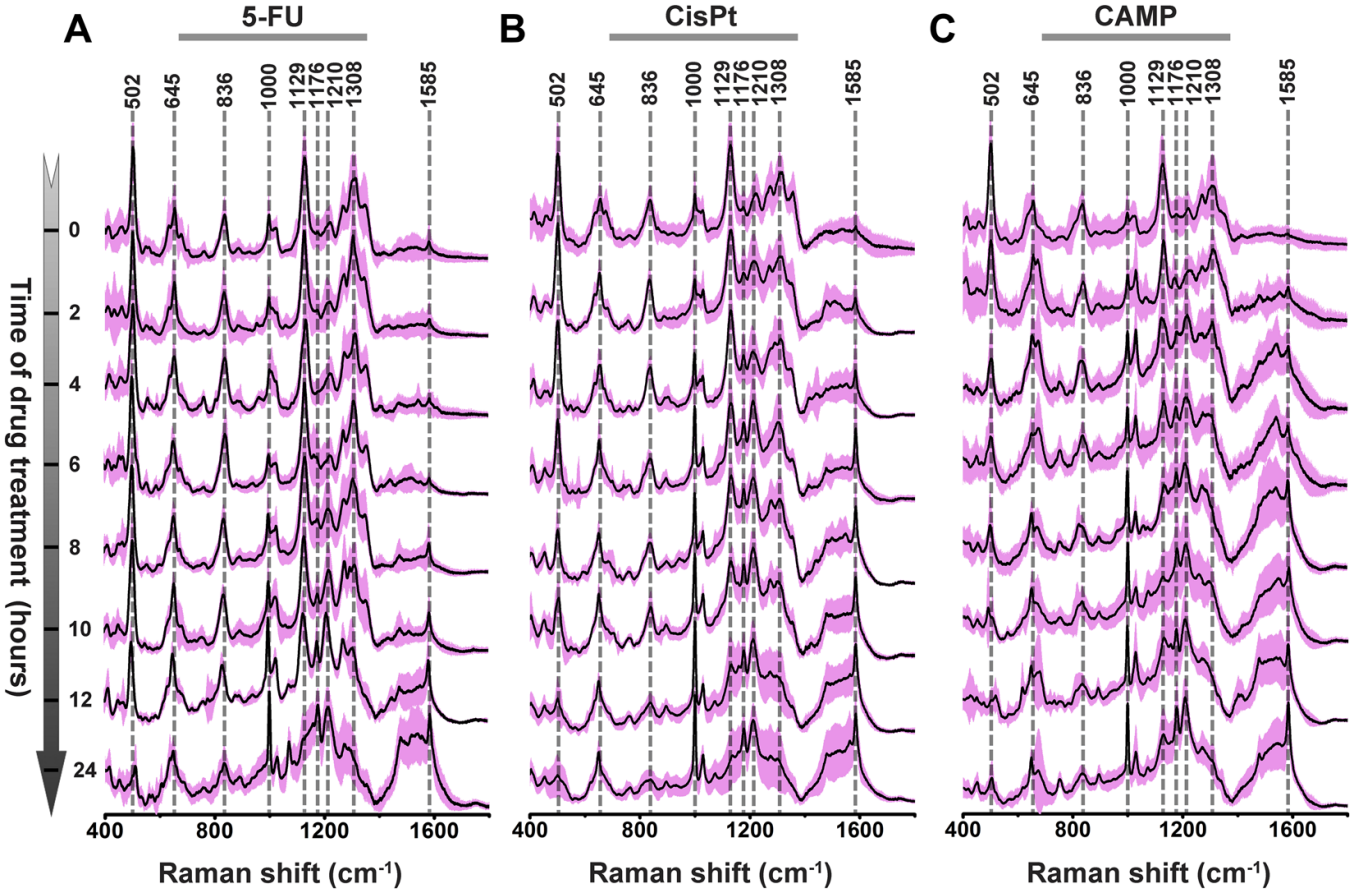
Real-time PERS spectra of cells under the treatment of 5-FU (**A**), CisPt (**B**) or CAMP (**C**). The averaged spectra from 30 trails of spectra were shown as black line and their standard deviation was highlighted as pink color.

Since the three drugs act on cell nucleus, we designed a nuclear targeting plasmonic nanoprobe to focus on the chemical modifications of proteins and nucleic acids at nuclear region. Nuclear targeting gold nanostars (NT-AuNSs), with size ~60 nm and very strong localized field at Raman incident wavelength (Fig. 1A and B), were utilized to target cell nucleus (see SI for details). Probe concentration was carefully controlled to minimize the effect to cell viability. After 24 h co-incubation, these NT-AuNSs were mainly localized around nuclear region (Fig. 1C and D). The presence of NT-AuNSs enhanced the Raman signals of cells for ~10^5^ folds, but none of the Raman bands of NT-AuNSs appeared as strong bands in the final cell PERS spectra (Fig. 1E). For studying the biomolecular modifications within MCF-7 cells during the processes of drug treatments, living cell PERS were carried out in presence of 5-FU, CisPt or CAMP. Raman spectra of control cells pretreated with 0.05 nM NT-AuNSs but without any drug treatment were collected to ensure cell viability and spectral reproducibility (Figures S6 and S7). Afterward, drug solutions (1 mM) diluted in complete culture media were passed through and the Raman spectra were collected at various time intervals from 10 different cells. The final acquired Raman spectra were averaged from three independent experiments. Real-time PERS spectra of cells under the treatment of the three drugs were shown in Fig. 2. We noticed several band changes along with drug treatments. Raman bands at 502 and 645 cm^−1^ are primarily attributed to S−S and C−S, respectively, from sulfur containing amino acids of proteins^25, 26^. The amide III bands of proteins appeared at 1210 cm^−1^ (amide III β-pleated sheet structure) and 1308 cm^−1^ (amide III α-helix conformation)^27, 28^. The vibrational band found at 1129 cm^−1^ is mainly composed of the C−N peptide bonds of proteins^12, 29, 30^. The band at 1000 cm^−1^ is attributed to phenylalanine^31, 32^. The C−H bending and C−C stretching of tyrosine were found at 1176 cm^−1^ ^33^. Band at 836 cm^−1^ is due to O−P−O backbone of DNA, and vibrations of guanine or adenine appeared at 1585 cm^−1^ ^14, 34–36^. A detailed tentative band assignments were given in Table S3. It is notable that since living cells were dynamic systems with multiple molecules, precision assignments of Raman bands to specific molecules were almost nearly impossible thus far. Even though, according to previous reports, some of the well-studied vibrational bands were assignable, some inconformity or even conflict were indeed excited in literatures. The dynamic changes on the features of Raman bands include substantial information about the chemical modifications during the processes of drug treatments, which offer the possibility of revealing the chemical paths and kinetics of drug-cell interaction through PERS spectra. To extract the most principal differences in chemical reaction paths among the three drug treated communities, we utilized PCA, which considers the main spectral features from multicomponent data sets (see SI for details). From the 2D scatter plots and 1D intensity plots (Figures S8 and S9), the classification of the three drug treated communities at each time point could be distinguished. It seems all these three drugs act on cells with quite similar “beginning” and “ending”, however, they undergo different processes.

### Chemical reaction kinetics of drug action

**Figure 3:**
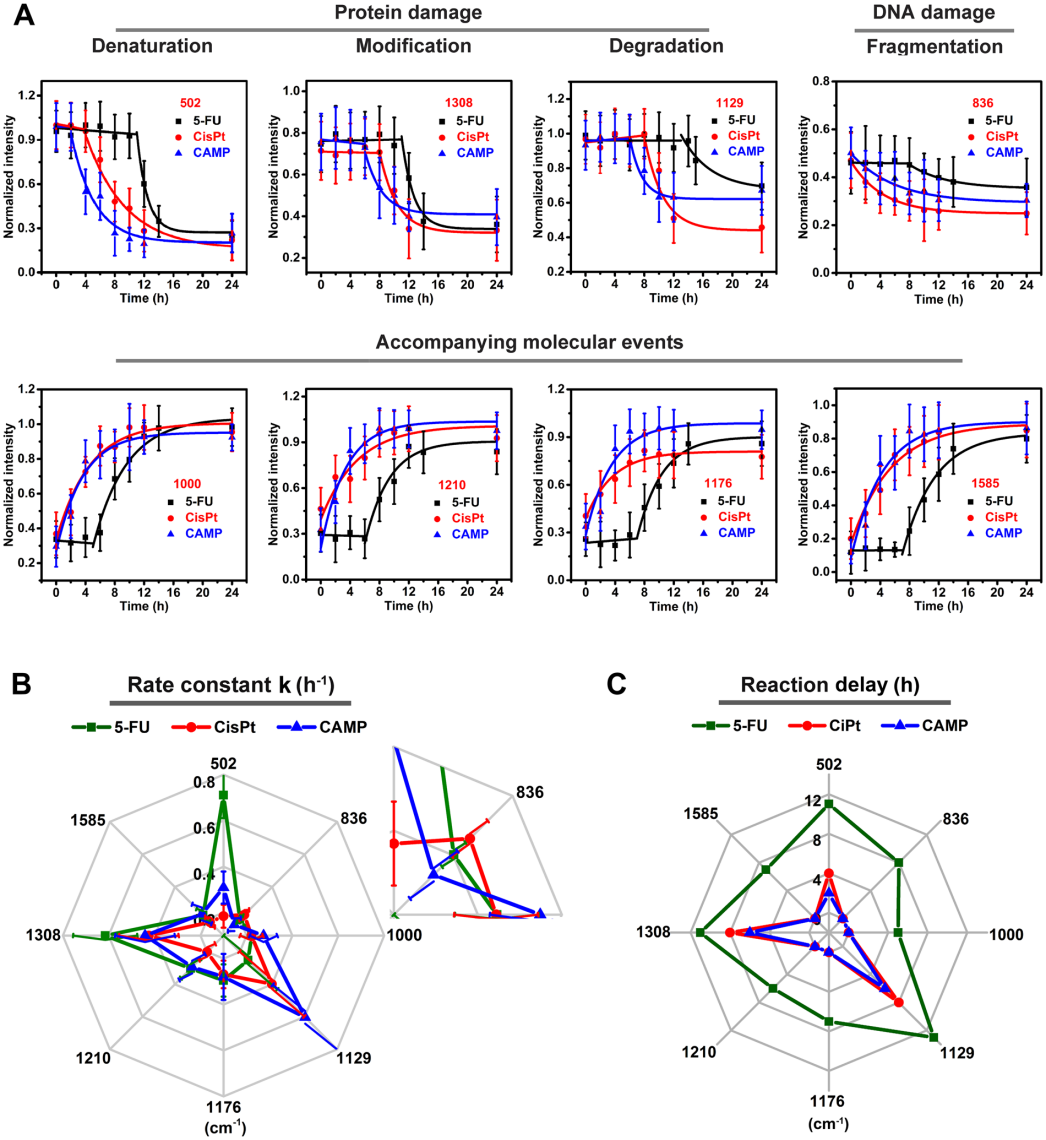
(**A**) Trends of the characterized Raman bands against time. Radar map of rate constant (**B**) and reaction delay (**C**).

To further understand the reaction kinetics of drug induced chemical changes within cells, we analyzed dynamic changes of Raman bands over time. Eight characteristic Raman bands were monitored and their intensities were well fitted with a first-order kinetic model (Fig. 3A). According to the fitting equation, the corresponding rate constants at different Raman shifts could be calculated (Table S4). The S−S band at 502 cm^−1^ showed a downward trend and achieved a balance in the end, indicating the rupture of disulfide bonds, i.e. the dissolution of tertiary protein structure^25, 26, 37^. We called this event protein denaturation. The amide III band at 1308 cm^−1^ (α-helix) also showed a decrease, which could be attributed to the conformational modification/misfolding of the proteins^19, 27, 28^. The C−N backbone band at 1129 cm^−1^ exhibited a downtrend, suggesting degradation of protein peptide bonds^12, 29, 30^. With the disruption of protein structure, hydrophobic amino acids, normally located in the interior of the protein, exposed from the folded structure^38, 39^. This was accompanied by the enhancement of the intensity of the 1000 (phenylalanine)^31, 32^, 1210 (β-pleated sheet)^27, 28^ and 1176 cm^−1^ bands (C−H, C−C of tyrosine)^33^, respectively. The decrease of 836 cm^−1^ band (O−P−O backbone) and increased 1585 cm^−1^ band (guanine/adenine) indicated DNA fragmentation^14, 18^, during which DNA lost its double stranded feature and allowing guanine or adenine to be exposed^40, 41^. These spectral changes demonstrated chemical bonds breakage, conformational modifications and damage of native structures in protein and DNA biomolecules within cells during drug treatment over time. For different drugs, the rate constants at different wavenumbers varied, and the reactions did not occur simultaneously (Table S5). Thus we defined a parameter, called reaction delay, to describe the time at which reactions started. To clearly see the difference, we illustrated the rate constants and reaction delay times as radar maps (Fig. 3B and C). Comparing three drugs, 5-FU had the largest rate constant at 502 and 1308 cm^−1^, while CisPt had the largest rate constant at 836 cm^−1^, and CAMP showed the largest rate constant at 1129 cm^−1^. In other words, 5-FU seems to mainly act on the S−S and amide III α-helix, CisPt acts on the O−P−O backbone of DNA, and CAMP primarily acts on the C−N peptide bond of protein. Besides, CisPt also had large rate constant at 1129 and 1308 cm^−1^, evidencing that CisPt not only acts on DNA but also works on protein. All of these results are basically in accordance with their action mechanisms. CisPt, as an important member of platinum-containing anticancer drugs, reacts in cells via binding to DNA and causing DNA damage, which ultimately triggers programmed cell death^42^. In this work, CisPt exhibited the largest rate constant at 836 cm^−1^, corresponding to direct and dominant DNA interaction. However, CAMP and 5-FU, which work through inhibition of DNA topoisomerase I and thymidylate synthase^43, 44^, showed large protein interaction rate constant (502, 1129, 1308 cm^−1^) instead of DNA interaction (836 cm^−1^). Even that, 5-FU tends to primarily cause protein denaturation (502 cm^−1^) and conformational modification (1308 cm^−1^), but CAMP mainly causes protein degradation (1129 cm^−1^). The reaction delay showed difference at different wavenumbers and depended on drugs. The reaction delay of 5-FU located at the outer ring compared with CAMP and CisPt, suggesting that reactions caused by 5-FU started slower than those of CAMP and CisPt. The rate constants and reaction delay times provided a new view point for discriminating drug action modes. It is pretty interesting to see that 5-FU, CisPt and CAMP reacted with proteins and nucleic acids through different paths, and induced chemical reactions with different kinetics. To confirm the occurrence of cellular apoptosis/necrosis and associated biomolecular events during the process of drug treatment, a series of control experiments were performed using traditional biological methods (see SI for details, Figure S10).

### Theoretical simulation

**Figure 4:**
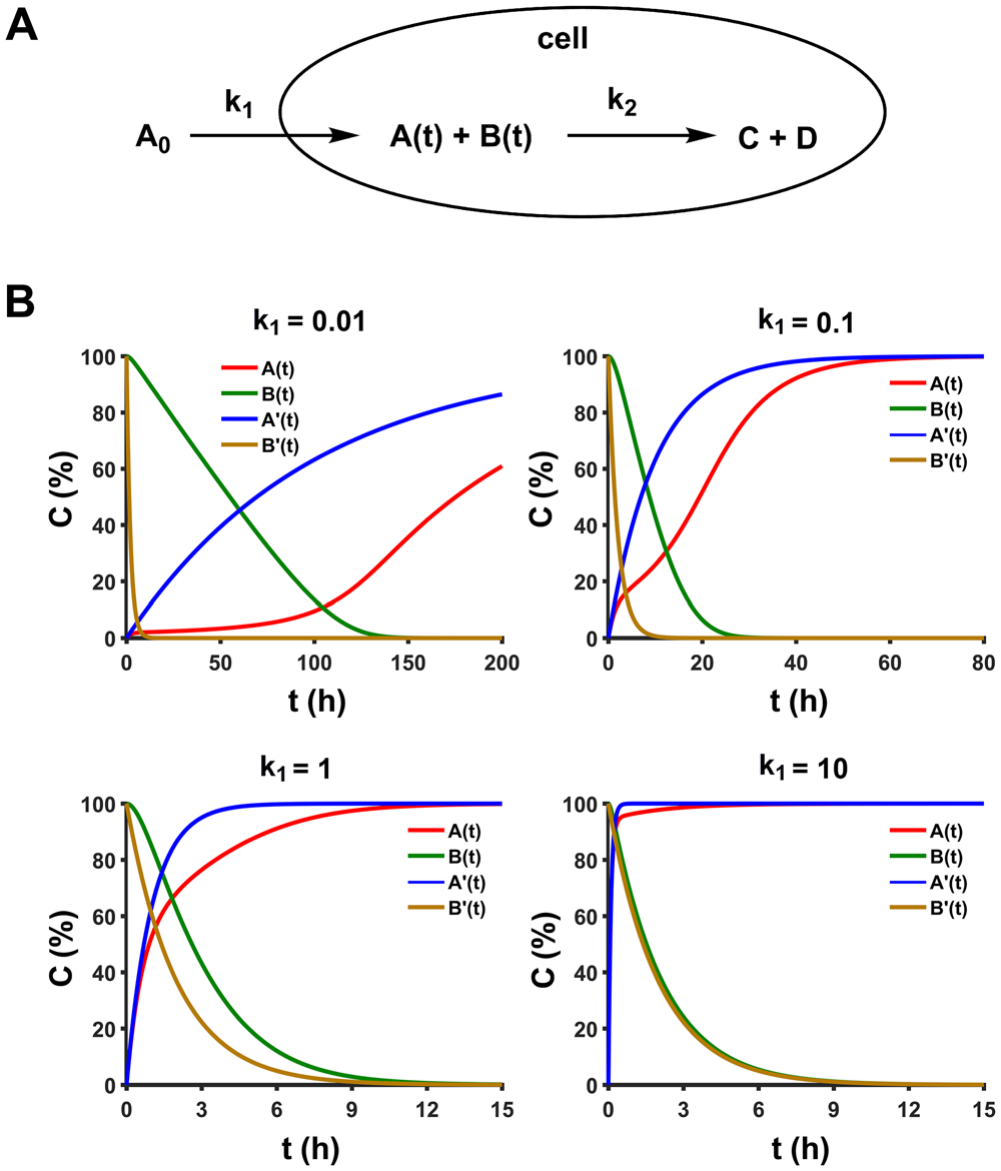
(**A**) Reaction model of drug uptake and action. (**B**) Theoretically simulation results at different initial *k*_*1*_ conditions (*k*_*2*_ = 0.5). *A*(*t)* and *B*(*t*) were normalized concentration of internal drug and biomolecules, respectively, *A*′(*t*) and *B*′(*t*) were their ideal exponential decay curves.

Besides the reaction kinetics, the reaction delay observed in this study is of great interest. To validate whether this delay originated from external drug uptake or internal molecular interaction, we developed a model to simulate this dynamical process (see experimental section and SI for details). We considered both the dynamic processes of drug uptake (with rate constant *k*_*1*_) and reaction with biomolecules (with rate constant *k*_*2*_), and investigated how they affected each other (Fig. 4A). We set *k*_2_ = 0.5 h^−1^, which is close to experimental observed value (for instance, at 1308 cm^−1^), changed *k*_*1*_ from conditions of *k*_*1*_ ≪ *k*_*2*_ (*k*_*1*_ = 0.01 h^−1^) to *k*_*1*_ ≫ *k*_*2*_ (*k*_*1*_ = 10 h^−1^), and then monitored normalized concentration of internal drug *A*(*t*) and biomolecules *B*(*t*), in comparison with their ideal exponential decay curves *A*′(*t*) and *B*′(*t*) (Fig. 4B). Results showed that, under all conditions from *k*_*1*_ ≪ *k*_*2*_ to *k*_*1*_ ≫ *k*_*2*_, we did not see delay curve as observed in experiments, which suggested that the delay was not from external uptake, but internal interaction. Also, at *k*_*1*_ ≪ *k*_*2*_ in which *B*(*t*) was dominated by *k*_*1*_, *B*(*t*) showed a decay curve mostly like linear response and with a decay time scale of 100 h, which is not the case we observed. When *k*_*1*_ changed from *k*_*1*_ ≪ *k*_*2*_ to *k*_*1*_ ≫ *k*_*2*_, *A*(*t*) and *B*(*t*) were close to their ideal exponential decay curves *A*′(*t*) and *B*′(*t*), and the decay time scale was also getting to comparable level with our experimental results. In our experiments, the decay curves of band change were fitted well by exponential first order rate equation. Put experimental and simulation results together, the reaction kinetic and delay observed here indeed originated from internal drug interactions, and the kinetic processes were dominated by internal interaction rate constants.

Up to now, some analytical methodologies for the determination of 5-FU, CiPt, and CAMP *in vitro*, such as UV-Vis Spectra, high-performance liquid chromatography (HPLC), Mass spectrometry (MS), and so on^45–48^, have been reported, but to the best of our knowledge, we did not find any related report about measuring the rate constants of drug reactions. Thus, the kinetic data presented in the paper would be very helpful for yielding better insight into drug action processes and mechanisms within single living cells.

## Conclusion

In summary, fully revealing the drug action processes and mechanisms within single living cells is a huge challenge and definitely have a long way to go. In this work, we tried to represent a small but vital step towards this goal. Even our current results were in infancy, it still could provide some helpful insight. As a proof-of-principle, we currently used three popular chemotherapy drugs as models. However, the strategy and methodology proposed here, have potentials to study the dynamic chemical reactions within cells under the treatments of any known or unknown drugs or related to various biological functions. Further efforts were expected to extend this method with more diversified probe design, more intelligentized data collection and more comprehensive data analysis, to resolve the dynamical chemical processes in single living cells with higher temporal, spatial and spectral resolutions.

## Methods

### Reagents

Tetrachloroauric acid trihydrate (HAuCl4 · 3H_2_O) was purchased from Sigma-Aldrich (Shanghai, China). Silver nitrate (AgNO_3_), ascorbic acid (AA), trisodium citrate, hydrochloric acid (HCl), fluorouracil (5-FU), cisplatin (CisPt), and camptothecin (CAMP) were purchased from Aladdin Chemistry Co. Ltd (Shanghai, China). Custom-made peptides such as nuclear localization signal (NLS, CGGGPKKKRKVGG) and cell penetrating peptide (RGD, RGDRGDRGDRGDPGC) were procured from Sangon Biotech (Shanghai, China). Thiol-modified methoxypolyethylene glycol (mPEG-SH, MW 5000) was obtained from Jenkem Technology Co. Ltd (Beijing, China).

### Instrumentation

Transmission electron microscopic (TEM) images of gold nanoparticles were obtained by a JEOL JEM-2100 microscope. UV-vis spectra were collected using a UV-3600 UV/Vis/NIR spectrometer. Dark-field (DF) images and scattering spectra were recorded on an inverted microscope (Olympus DP80) combined with a Princeton Instruments grating spectrometer. Hydrodynamic diameters were confirmed through a BI-90Plus dynamic light scattering spectrometer. Raman spectra were collected using a Renishaw inVia-Reflex Raman spectrometer, equipped with a 785 nm excitation laser, and a microscope with a 50 ×/0.5 N.A. objective lens.

### Synthesis of gold nanostars (AuNSs)

AuNSs were prepared by a seed-mediated growth method according to a modified version of the surfactant-free nanostar synthesis described by Vo-Dinh *et al*.^49^. For seed preparation, 15 mL of 1% trisodium citrate solution was added to 100 mL of boiling 1 mM HAuCl_4_ solution under vigorous stirring. After 15 min of boiling, the solution was cooled under mild stirring, and then kept at 4 °C for long-term storage. For nanostar synthesis, 0.5 mL of the above seed solution (~13 nm, *A* = 2.81) and 50 μL of 1 M HCl were added to 50 mL of 0.25 mM HAuCl_4_ solution at room temperature under moderate stirring. Quickly, 1 mL AgNO_3_ of different concentrations (0.125, 0.25, 0.5, 1, and 2 mM) and 0.25 mL of 0.1 M AA were added simultaneously. The solution was stirred for 30 s, and then kept at 4 °C for long-term storage.

### Preparation of nuclear targeting gold nanostars (NT-AuNSs)

The AuNSs (50 mL of 0.1 nM) were first incubated with 100 μL of 0.05 mM mPEG-SH for 12 h. This solution was purified by centrifugation at 8000 rpm for 10 min. Subsequently, these PEGylated AuNSs (4 mL of 0.5 nM) were treated with 40 μL of 0.5 mM RGD, and 400 μL of 0.5 mM NLS, to yield nuclear targeted AuNSs (NT-AuNSs). These NT-AuNSs were purified using centrifugation at 8000 rpm for 10 min to remove unbound ligands and were redispersed in deionized water for subsequent use.

### Cell Culture

MCF-7 cells (human breast adenocarcinoma cell line) were cultured in Dulbecco’s modified Eagles’ medium (DMEM, KeyGEN BioTECH), which contains phenol red, supplemented with 4.5 g/L glucose and sodium pyruvate, 10% fetal bovine serum (FBS, Life), and 1% antimycotic solution (KeyGEN BioTECH) at 37 °C in a 5% CO_2_ humidified incubator.

### Anticancer drug treatment and PERS measurement

Briefly, MCF-7 cells were cultured on 18 mm coverslips in complete growth medium in incubator at 37 °C overnight. Cells were then treated with 0.05 nM NT-AuNSs, diluted in complete cell culture medium, for 24 h. After NT-AuNSs treatment, the coverslips were placed in a homemade live cell chamber that maintained stable humidity, 37 °C temperature and 5% CO_2_ concentration. Drug solutions (1 mM) diluted in complete culture media were injected into the live cell chamber using an auto-injection system and Raman spectra were obtained over 24 h. The pretreatment with NT-AuNSs enabled acquisition times of well-resolved spectra to be 10 s using the extended scan mode. Raman spectra from three independent experiments were averaged and normalized to the most intense band.

### Data analysis

Spectral data from our experiments were processed and analyzed using MATLAB R2015b. Principle component analysis (PCA) was utilized for data analysis as it allows determination of subtle differences within multidimensional data sets. Prior to PCA, 20 of spectra from each group (treated by three drugs) were randomly selected and normalized to [0, 1]. The scatter plot of PC1 vs PC2 scores were utilized to classify the three drug treated communities. A Kernel density estimation method was used to smooth the diagram of scores to generate a 1D intensity distribution. The rate constants were obtained by fitting the dynamic changes of Raman bands with a first order kinetic model.

### Finite-difference time-domain (FDTD) simulation

The electromagnetic field distributions of metal nanostructures were simulated by the FDTD method via a commercial software package (Lumerical Solutions, Inc.). The dielectric constant of gold was from John and Christy^50^. The computational domain was bounded by a perfectly matched layer (PML) to prevent any reflections back onto the nanoparticle. The nanoparticle was excited with a quasi nonpolarized light, consisted of x-polarized and y-polarized incident plane wave with E-field amplitude 1, propagating along the z-axis. The mesh unit was 1 × 1 × 1 nm^3^.

### Flow cytometry

Apoptotic cell death was detected by staining with Annexin V/propidium iodide (PI) (Thermo Fisher Scientific) according to the manufacturer’s protocols. In brief, cells were harvested and washed once with phosphate buffered saline (PBS), then resuspended in 100 uL binding buffer followed by incubation with 2.5 uL Annexin V per test for 20 min. Then, 1 uL PI per test was added and stained cells were analyzed by a FACSCalibur cytometer (Becton, Dickinson and Company, Franklin Lakes, NJ). Data were analyzed using FlowJo software (Version 7.6.5, Tree Star, Ashland, OR).

### Confocal microscopy

Treated cells were stained with 4′, 6-diamidino-2-phenylindole (DAPI) after fixed with 4% paraformaldehyde. Cells were observed under FLUOVIEW FV10i confocal microscope (Olympus, Tokyo, Japan) and images were analyzed using FV10-ASW 4.0 Viewer (Olympus).

### Western blot

Cells were lysed in radio-immunoprecipitation assay (RIPA) buffer containing a protease inhibitor cocktail (Roche, Mannheim, Germany, 11873580001). Protein concentration was determined. Equal amounts of protein were separated by sodium dodecyl sulfate-polyacrylamide gel electrophoresis (SDS-PAGE) and electrophoretically transferred onto a poly(vinylidene fluoride) (PVDF) membrane (Roche, 03010040001). After blocking with 5% nonfat milk in Tris-buffered saline containing 0.1% Tween-20 the membrane was incubated with specific primary antibodies, followed by incubation with appropriate horseradish peroxidase (HRP)-conjugated secondary antibodies. Signals were detected using an enhanced chemiluminescence reagent (Millipore, Darmstadt, Germany, WBKLS0500) and subjected to chemiluminescence instrument (Beijing Sage Creation Science Co. Ltd). Poly ADP-ribose polymerase (PARP) antibody (Promega, Madison, WI, G734A, 1:1000 dilution), Glyceraldehyde 3-phosphate dehydrogenase (GAPDH) antibody (Bioworld, Minneapolis, MN, MB001, 1:5000 dilution), HRP-conjugated secondary antibodies (Multisciences, Hangzhou, China, GAR007 and GAM007, 1:5000 dilution).

### Mathematic modeling and calculation

Considering both the external drug uptake and internal drug action, we have built a model to study the dynamic processes of drug uptake (with rate constant *k*_*1*_) and reaction with biomolecules (with rate constant *k*_*2*_) as shown in Fig. 4A. The reaction rate of *A*(*t*) and *B*(*t*) can be expressed with

1 $$\frac{dA(t)}{dt}={k}_{1}({A}_{0}-A(t))-{k}_{2}\,\ast \,A(t)\,\ast \,B(t)$$

2 $$\frac{dB(t)}{dt}=-\,{k}_{2}\,\ast \,A(t)\,\ast \,B(t)$$

Instead of analytically solving *A*(*t*) and *B*(*t*) from above two equations, which are pretty difficult to solve, we proposed a model to effectively calculate the numerical solution of A and B at any time point of t. See SI for calculation details.

## Electronic supplementary material

Supporting Information
