# Supplementary material for: Revealing chemical processes and kinetics of drug action within single living cells via plasmonic Raman probes
Source: Sci Rep. 2017 May 23;7:2296. doi: 10.1038/s41598-017-02510-9 (PMC5442120; doi:10.1038/s41598-017-02510-9)
Supplement: Supplementary file 1 — Supporting Information [file 41598_2017_2510_MOESM1_ESM.doc]

**Supplementary Information**

Revealing chemical processes and kinetics of drug action within single living cells via plasmonic Raman probes

Shan-Shan Li^1^, Qi-Yuan Guan^1^, Gang Meng^2^, Xiao-Feng Chang^3^, Ji-Wu Wei^2^, Peng Wang^3^, Bin Kang^1,*^, Jing-Juan Xu^1,*^, and Hong-Yuan Chen^1,*^

^1^State Key Laboratory of Analytical Chemistry for Life Science and Collaborative Innovation Center of Chemistry for Life Sciences, School of Chemistry and Chemical Engineering, Nanjing University, 210023, China

^2^Jiangsu Key Laboratory of Molecular Medicine, Medical School and the State Key Laboratory of Pharmaceutical Biotechnology, Nanjing University, 210093, China

^3^National Laboratory of Solid State Microstructures, College of Engineering and Applied Sciences and Collaborative Innovation Center of Advanced Microstructures, Nanjing University, Nanjing 210093, China

1. **Additional figures, tables and discussions**


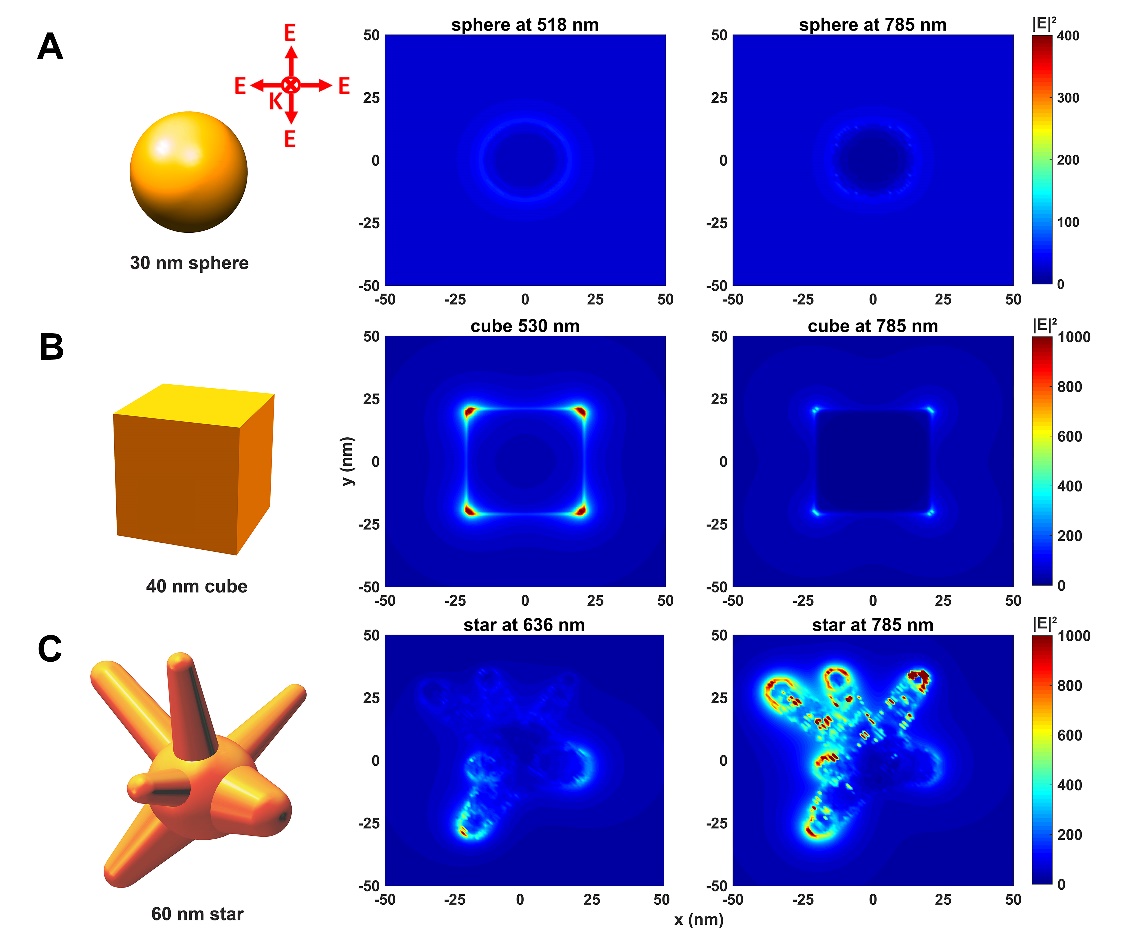


**Figure S1** Design of the nuclear-targeted plasmonic nanoprobes. (A) FDTD simulated distribution of localized electronic field of gold nanosphere (A), nanocube (B) and nanostar (C) at their maximum extinction wavelength and Raman excitation wavelength.

Several types of plasmonic nanoprobes, such as gold nanospheres and nanocubes[^1-4^](#_ENREF_1), have been used to target to specific locations inside cells for enhancing the Raman signals. Herein, we used gold nanostars (AuNSs), as the localized electric field of AuNSs was much higher than that of nanospheres or nanocubes at 785 nm excitation laser (Fig. S1). According to PERS theory, the signal intensity is proportional to |*E*_loc_/*E*_in_|^4^, where *E*_loc_ and *E*_in_ are the amplitudes of the localized electric field at the surface of probes and incident field, respectively[^5^](#_ENREF_5). Thus theoretically, AuNSs can enhance the intensities of Raman vibrations to a larger extent, compared to spherical or cubic probes.


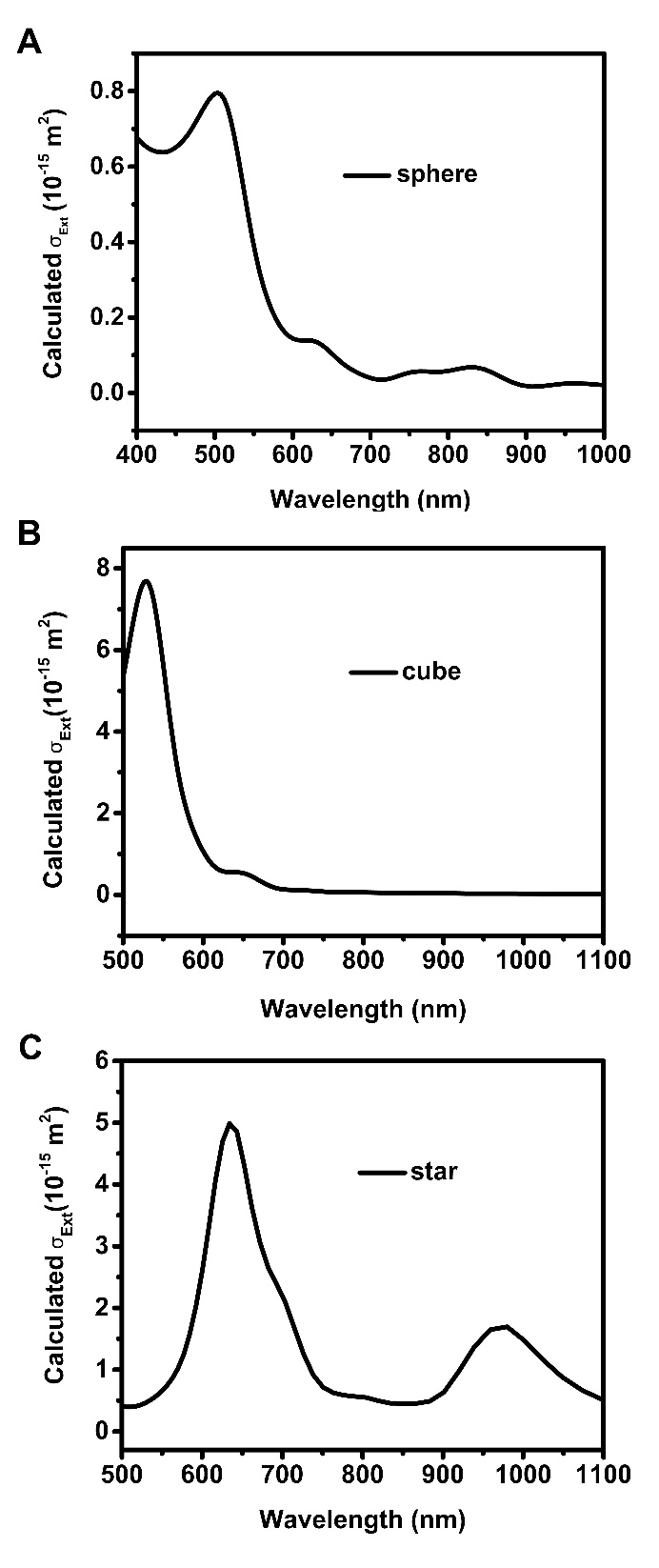


**Figure S2** FDTD calculated extinction spectra of gold nanosphere (A), nanocube (B) and nanostar (C).


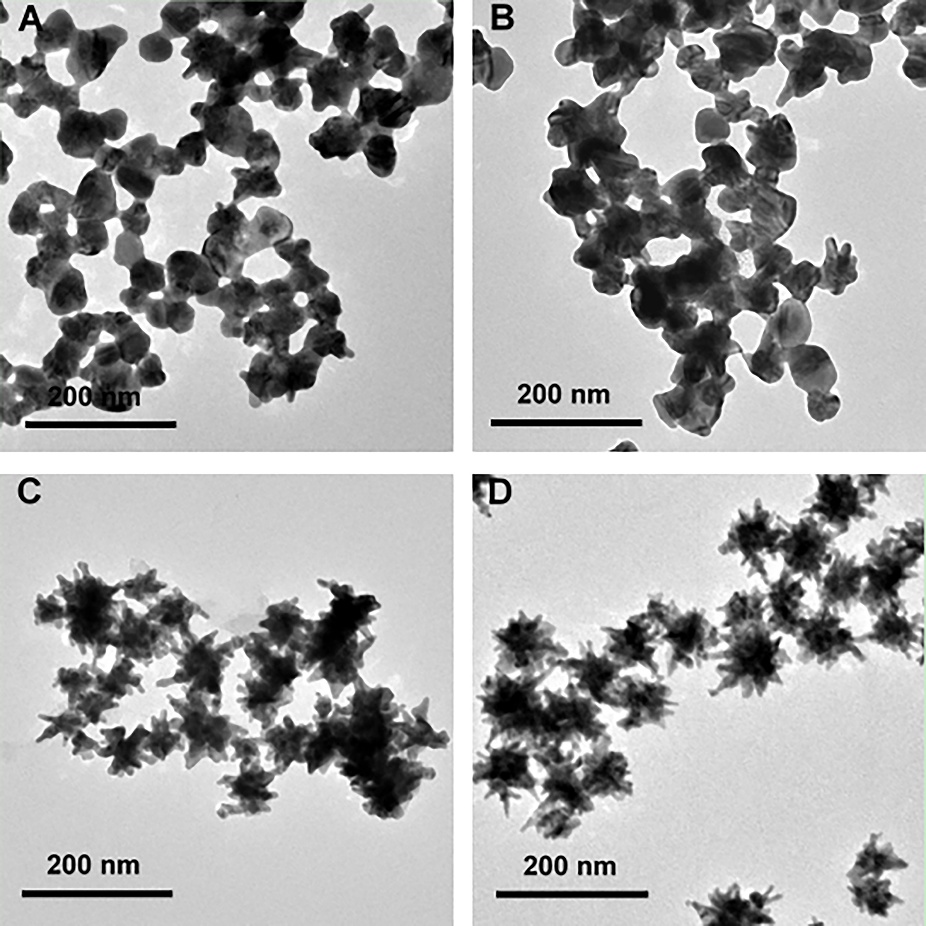


**Figure S3** TEM micrographs of AuNSs obtained with 2.5 (A), 5 (B), 20 (C), and 40 (D) μM AgNO_3_.

These AuNSs were synthesized following a surfactant-free method, where AgNO_3_ acted as shape-directing agent to complete nanocrystal growth[^6^](#_ENREF_6). By changing the concentrations of AgNO_3_, the morphology of AuNSs could be tailored (Figure S3, S4 and Table S1).


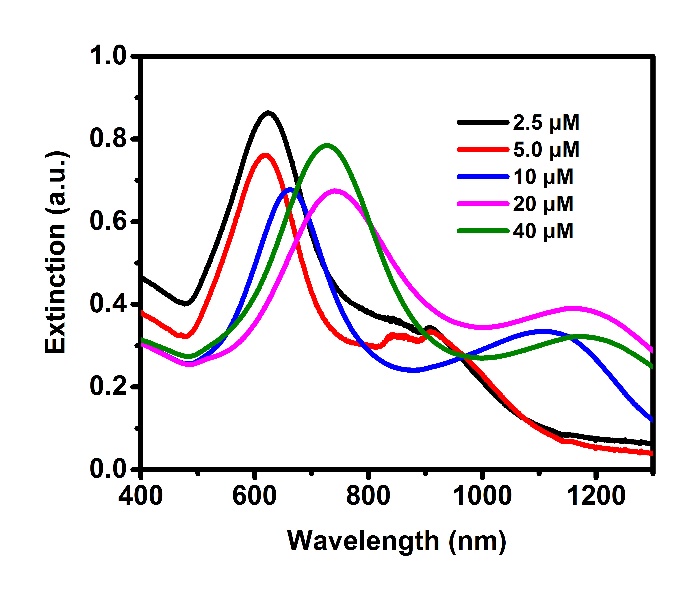


**Figure S4** Extinction spectra of AuNSs obtained with different concentrations of AgNO_3_.


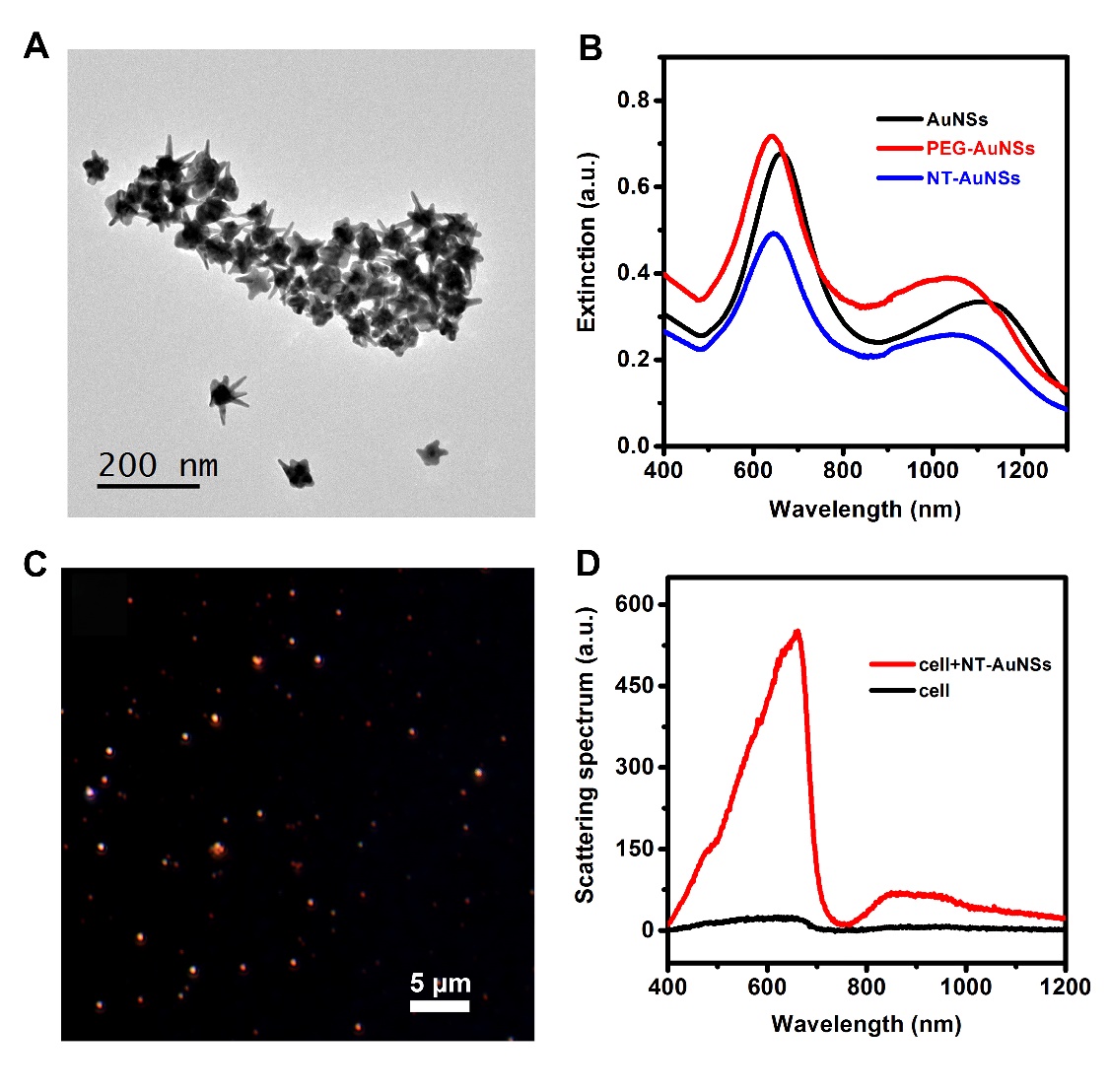


**Figure S5** (A) TEM micrograph of AuNSs. (B) UV-Vis extinction spectra of AuNSs before (black) and after conjugation with PEG (red), and RGD and NLS peptides (blue) in aqueous solution. (C) Dark field scattering image of AuNSs. (D) The corresponding scattering spectra of cells with (red) and without (black) NT-AuNSs.

The morphology of optimized AuNSs for plamonic probes was shown in Figure S5A, with an average size of 60 nm. Such AuNSs were functionalized with thiol-modified methoxypolyethylene glycol (mPEG-SH) molecules, in order to ensure their stability in biological environments and inhibit nonspecific binding of proteins[^7^](#_ENREF_7). Then, the AuNSs were further functionalized with RGD peptide (targeting *αβ* integrins on the MCF-7 cell membrane to assist the uptake of AuNSs) and nuclear localization signaling (NLS, which targets the AuNSs to the nuclear region of MCF-7 cells). RGD and NLS conjugated nanoparticles can target to the nuclear region of various cells. Successful conjugation was confirmed through the little shifts of extinction spectra (Figure S5B) and the changes in hydrodynamic diameters obtained via dynamic light scattering (Table S2). The AuNSs showed bright orange red Rayleigh/Mie scattering under dark-field microscope (Figure S5C). Besides, the scattering spectrum of NT-AuNSs treated cell was much stronger than that of control cell (Figure S5D).


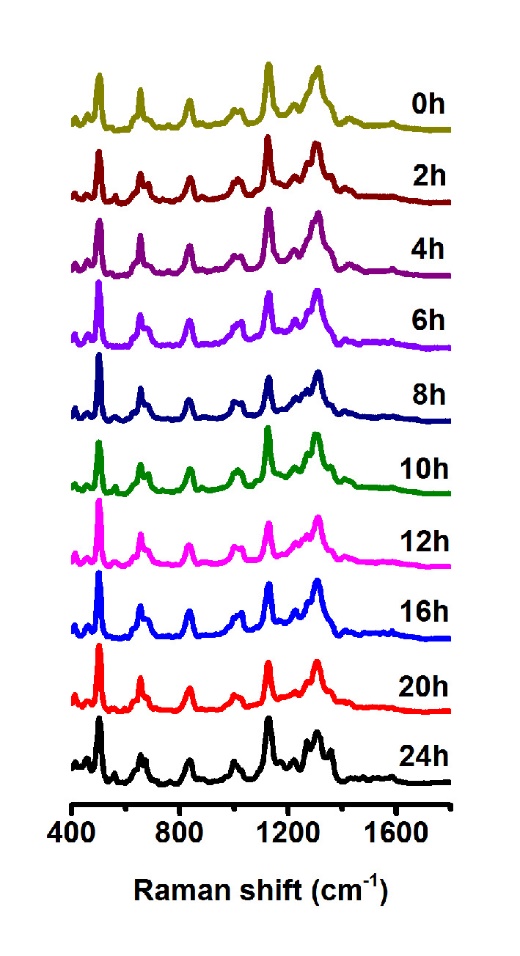


**Figure S6** Real-time PERS spectra of control MCF-7 cells without any drug treatment.


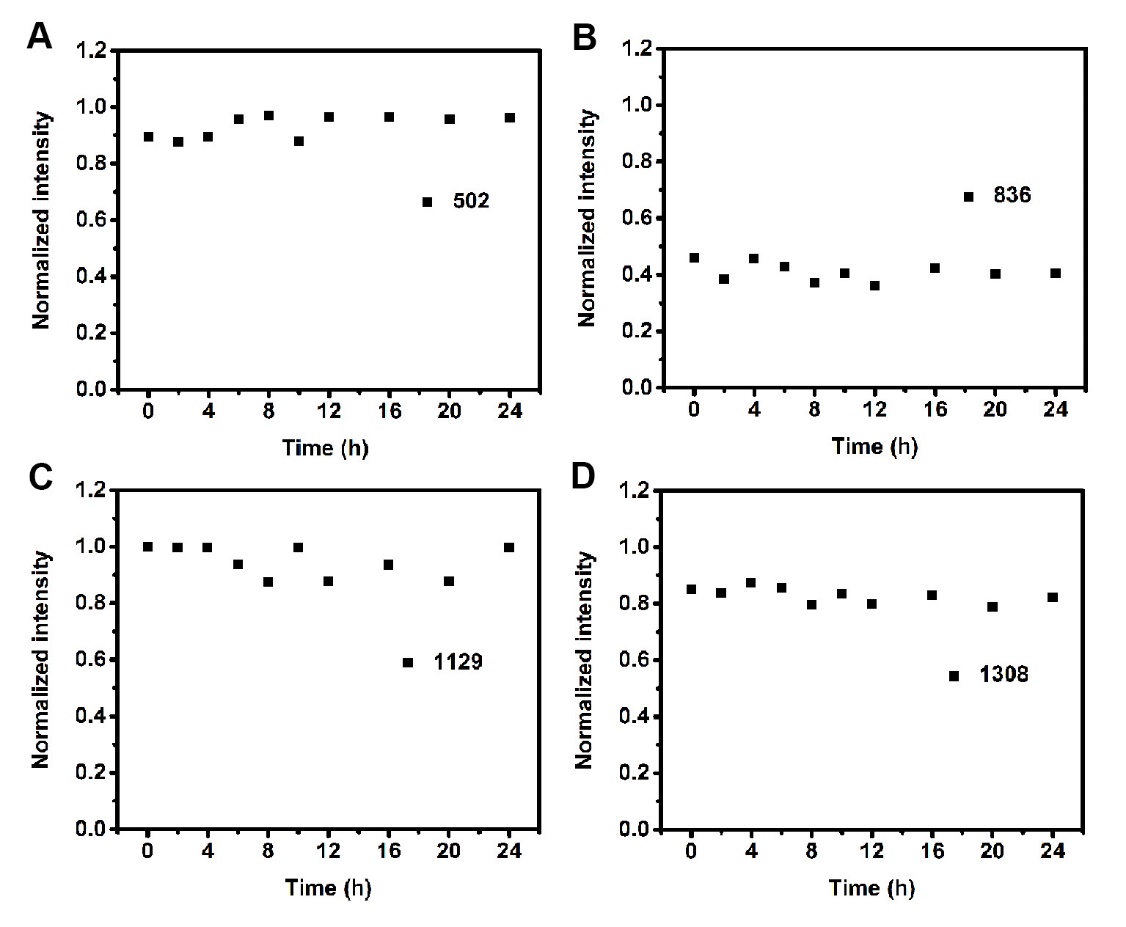


**Figure S7** The characterized Raman bands of control MCF-7 cells against time.

Real-time PERS experiments were perform on controlled cells pretreated with 0.05 nM NT-AuNSs for 24 h, but without any drug treatments (same amount of culture medium was added for control, Figure S7). Results showed that the Raman spectra of cells remain unchanged within 24 hours, which clearly indicated that the plasmonic nanoprobes at such low concentration rarely affect the cell viability and intracellular molecular signatures. It also proved that our on-stage cell chamber could provide proper environment to keep cell alive during the period of our experiments. These results also demonstrated spectral stability during the periods of cell growth, which also confirmed that the band changes observed were indeed from drug action.


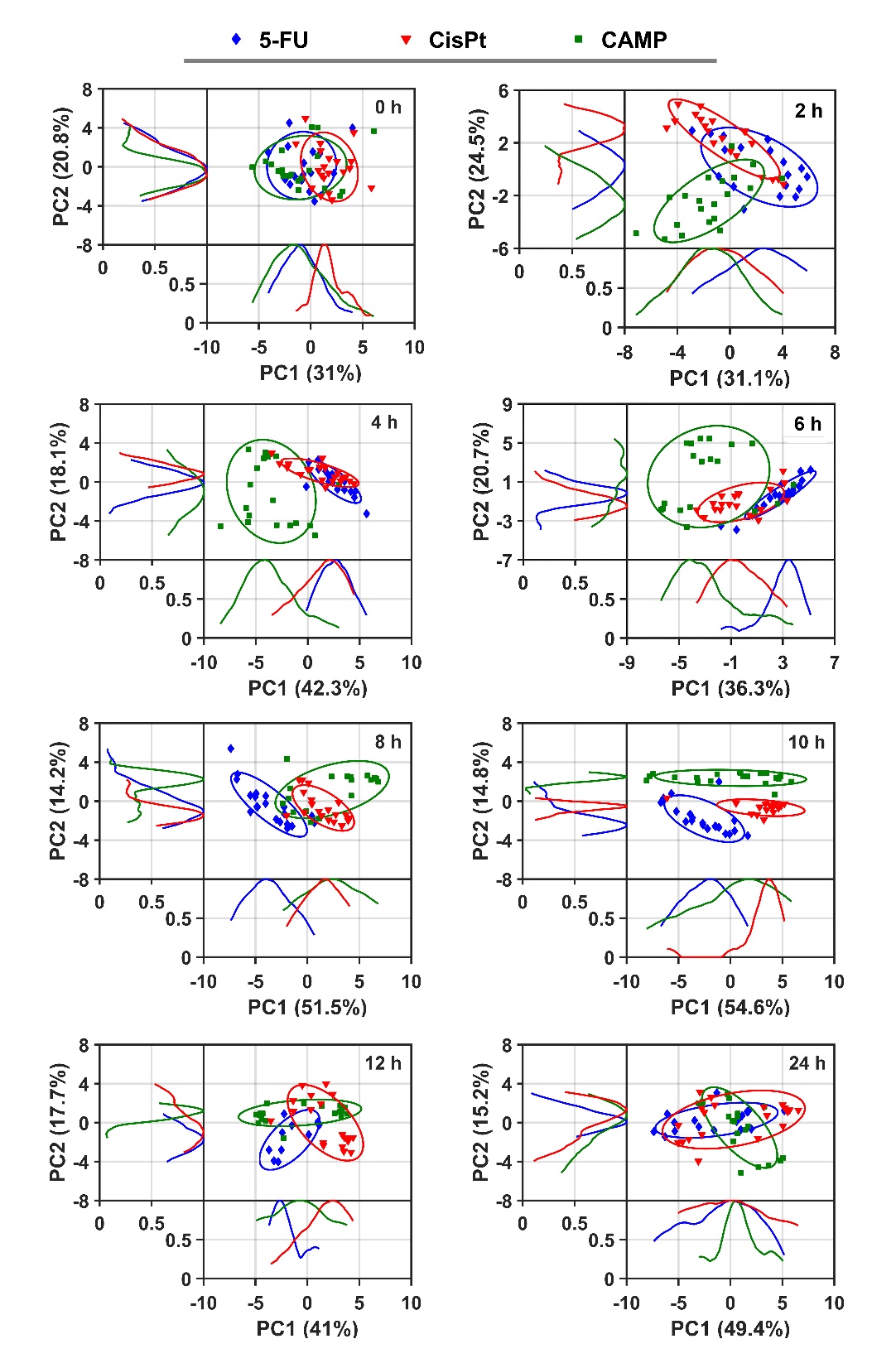


**Figure S8** PCA of drug action paths. Scatter plot of PC1 vs PC2 scores with attached 1D intensity lines of the PERS spectra at different time point of drug treatment.

In order to extract the most principal differences in chemical reaction paths among the three drug treated communities, we utilized a low-level correlation based method, principal component analysis (PCA), which considers the main spectral features from multicomponent data sets. PERS data sets (20 data points each) for three drug treated communities at each time point were analyzed using PCA and the results were shown as 2D scatter plots for PC scores (Figure S8). The 1D intensity plots are smoothed histograms of the PC data and allow for easy characterization of the data distribution. From the 2D scatter plots and 1D intensity plots, the classification of the three drug treated communities at each time point could be distinguished (Figure S8). It is interesting to note that at the early stage of drug action (0 h) or at very late stage (24 h), the three cell communities treated by 5-FU, Cispt and CAMP basically superposed together. However, during the middle time points of drug treatment process, the three cell communities could be clearly distinguished (8-12 h), with the most significant discrimination at 10 h. These results suggested that at the beginning or nearly ending of drug treatment, the spectra of these three cell communities were very similar. With the increase of drug treatment time, cells treated by different drugs underwent different chemical evolutions, resulting in the different communities in 2D PC scores plots. It seems all these three drugs act on cells with same beginning and same ending, however, they undergo different paths.


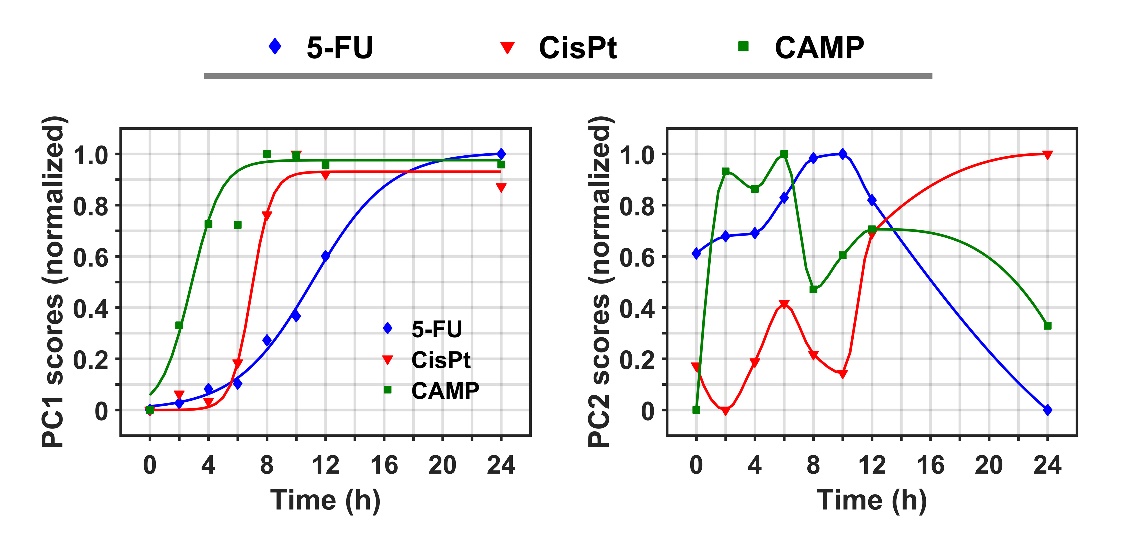


**Figure S9** Trends of averaged PC1 and PC2 scores against time during the period of drug treatment.

To clearly distinguish different action paths of these three drugs, the normalized intensity of PC scores were plotted out over treatment time (Figure S9). The PC1 scores showed sigmate response curves for all three drugs over time of treatment, while PC2 scores of these three drugs separated well during all period of treatment. It might be because that the PC1 scores are the most principal components, which indicate the primary difference on the cell communities treated by three types of drugs. PC2 scores, as the secondary components with lower weight, show more detailed difference among such three communities. To concisely compare the dynamical processes of chemical changes caused by these three drugs, we defined an index, termed half-effect time T-50, which means the time when PC1 scores changed up to 50%. The T-50 for these three drugs were 11 h (5-FU), 7 h (CisPt) and 3 h (CAMP), respectively. It is notable that the T-50 in current work was defined as the time of 50% PC1 scores changes happened, which indicated the efficacy of drug induced chemical modifications. The value of T-50 might be related to, but not have to be consistent with the actual therapeutic efficacy of drugs. Therefore, the most significant differences in chemical reaction paths among the three drug treated communities can be picked out by PCA analysis.


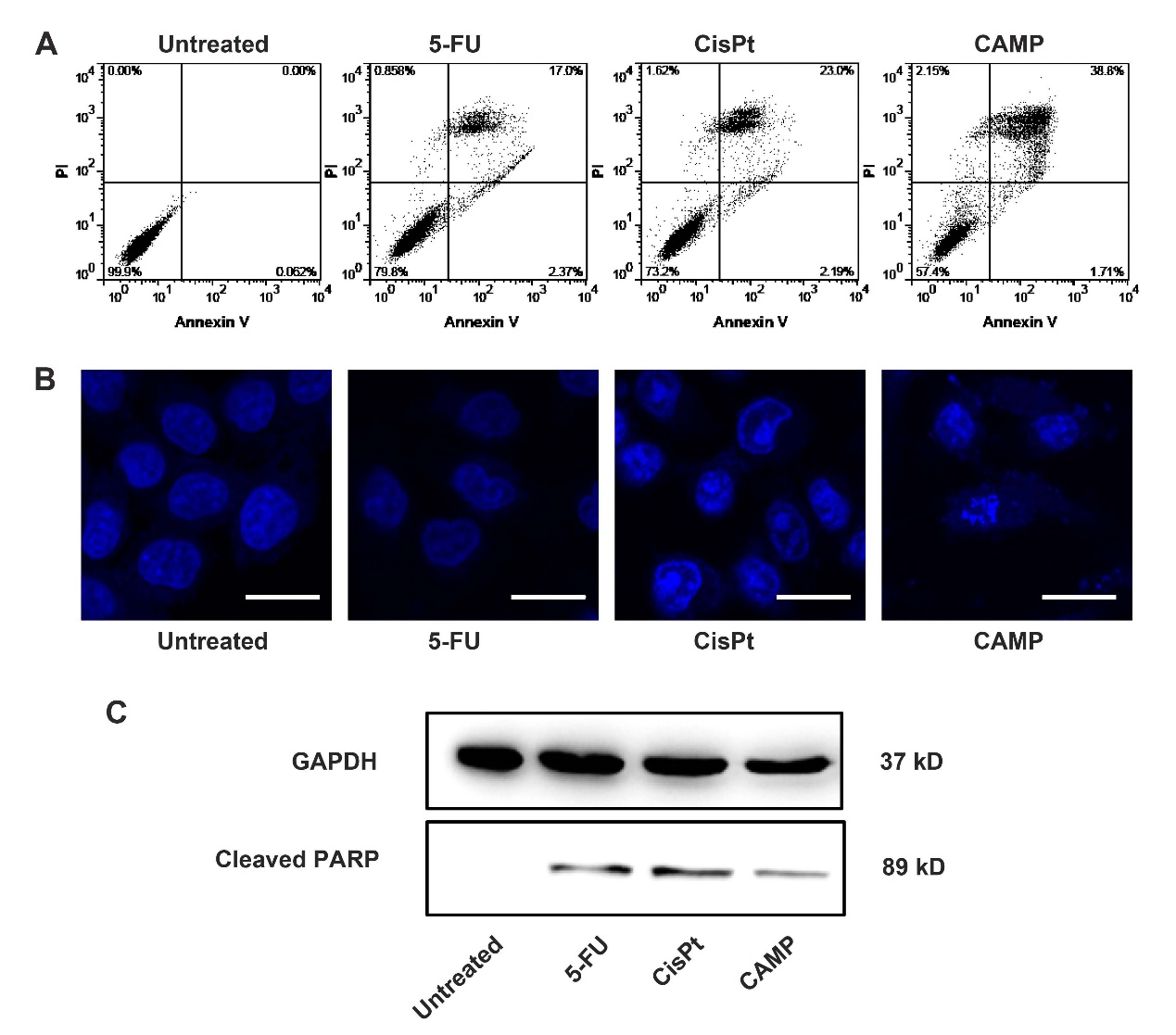


**Figure S10** Drug induced cell apoptosis, nuclear fragmentation and DNA damage. (A) Flow cytometry of MCF-7 cells treated with 5-FU, CisPt or CAMP, cells were stained with Annexin V-FITC and propidium iodide (PI). (B) Confocal microscope of the nuclei of MCF-7 cells after treatments with 1 mM 5-FU, CisPt or CAMP for 24h. Cell nuclei were stained with 4', 6-diamidino-2-phenylindole (DAPI). The scale bar is 20 μm. (C) Western blot of MCF-7 cells treated with 5-FU, CisPt or CAMP showing activation of Poly (ADP-ribose) polymerase (PARP).

To confirm the occurrence of cellular apoptosis/necrosis and associated biomolecular events during the process of drug treatment, a series of control experiments were performed using traditional biological methods, i.e. flow cytometry, fluorescence microscopy and western blot, as shown in Figure S10. Flow cytometry of cell apoptosis/necrosis array indicated that cell groups treated by all these three drugs underwent late apoptosis or necrosis (Figure S10A). We also monitored nuclear fragmentations by 4',6-diamidino-2-phenylindole (DAPI) staining in drug treated cells (Figure S10B), Cispt and CAMP treatments induced relative strong nuclear fragmentations and 5-FU induced relative weak fragmentations, which basically consisted with results obtained by flow cytometry as well as our PERS-metrics. Furthermore, we detected activation of poly (ADP-ribose) polymerase (PARP) by western blot in drug treated cells. PARP is an enzyme involving DNA damage and program cell death. It helps cells to maintain their viability, and the cleavage of PARP facilitates cellular disassembly and serves as a marker of cells undergoing apoptosis[^13^](#_ENREF_13). Results indicate that drug treatment induced the cleavage of PARP (Figure S10C), which suggests that treatments of all three drugs increased cellular DNA damage in MCF-7 cells.

**Table S1.** Hydrodynamic diameter of synthesized AuNSs with different concentrations of AgNO_3_.

| Concentration of AgNO_3_ (μM) | Hydrodynamic diameter (nm) | Polydispersity |
| --- | --- | --- |
| 5 | 29.02±2.39 | 0.58 |
| 10 | 52.78±0.58 | 0.32 |
| 20 | 78.07±0.48 | 0.24 |
| 40 | 76.76±0.86 | 0.23 |

**Table S2.** Hydrodynamic diameter of AuNSs, PEG-AuNSs and NT-AuNSs.

| Sample | Hydrodynamic diameter (nm) | Polydispersity | |
| --- | --- | --- | --- |
| AuNSs | 52.78±0.58 | | 0.32 |
| PEG-AuNSs | 72.15±0.28 | | 0.31 |
| NT-AuNSs | 69.21±1.61 | | 0.32 |

**Table S3.** Tentative assignments of the characterized bands in PERS spectra.

| Band (cm^-1^) | Component | Tentative assignment of PERS bands |
| --- | --- | --- |
| 502 | Protein | S−S stretching |
| 645 | Protein | C−S stretching |
| 836 | DNA | backbone, O−P−O stretching |
| 1000 | Protein | ring breathing of phenylalanine |
| 1129 | Protein/lipid | C−N stretching and lipid all-trans conformations |
| 1176 | Protein | C−H bending and C−C stretching of tyrosine |
| 1210 | Protein | amide III (*β*-pleated sheet) |
| 1308 | Protein | amide III (*α*-helix) |
| 1585 | DNA/protein | guanine/adenine, phenylalanine |

**Table S4.** Rate constants at different wavenumbers in cells.

| Band (cm^-1^) | Rate constant k (h^-1^) | | |
| --- | --- | --- | --- |
|  | 5-FU | CisPt | CAMP |
| 502 | 0.71±0.10 | 0.18±0.05 | 0.31±0.07 |
| 836 | 0.20±0.02 | 0.23±0.03 | 0.17±0.04 |
| 1000 | 0.22±0.05 | 0.22±0.05 | 0.27±0.06 |
| 1129 | 0.25±0.18 | 0.40±0.20 | 0.60±0.20 |
| 1176 | 0.30±0.07 | 0.27±0.06 | 0.28±0.10 |
| 1210 | 0.30±0.06 | 0.20±0.05 | 0.29±0.08 |
| 1308 | 0.62±0.14 | 0.43±0.13 | 0.44±0.13 |
| 1585 | 0.23±0.04 | 0.18±0.03 | 0.23±0.06 |

**Table S5.** Reaction delay times at different wavenumbers in cells.

| Band (cm^-1^) | Reaction delay (h) | | |
| --- | --- | --- | --- |
|  | 5-FU | CisPt | CAMP |
| 502 | 11 | 4 | 2 |
| 836 | 8 | 0 | 0 |
| 1000 | 5 | 0 | 0 |
| 1129 | 13 | 8 | 6 |
| 1176 | 7 | 0 | 0 |
| 1210 | 6 | 0 | 0 |
| 1308 | 11 | 8 | 6 |
| 1585 | 7 | 0 | 0 |

1. **Mathematic modeling and calculation**

We have built a model to study the dynamic processes of drug uptake and reaction with biomolecules depicted below:


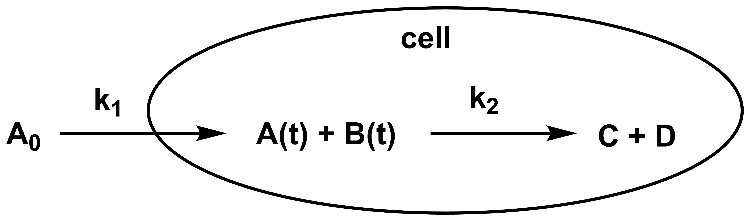


The reaction rate of *A(t)* and *B(t)* can be expressed with


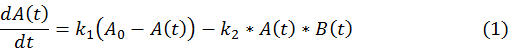


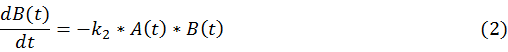


Instead of analytically solving *A(t)* and *B(t)* from above two equations, which are pretty difficult to solve, we proposed a model to effectively calculate the numerical solution of *A* and *B* at any time point of *t*.

As an initiative condition for this reaction, the concentration of drug *A* outside the cell and internal molecular *B* were both set to 1, and the concentration of drug *A* inside the cell was set to 0 at the beginning of the reaction. The reaction can be separated into two steps alternately: drug *A* diffuses inward and *A* inside the cell reacts with molecular *B*. At *t*=0, we have


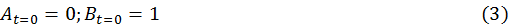


After a very short time period *ΔT*, the rate of *A* transported inward the cell can be considered uniformed. We can write it as


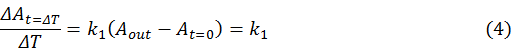


where *ΔA_t=ΔT_* is amount change of *A* during this time period *ΔT* when time *t=ΔT*, *k_1_* is the diffusion coefficient of *A* and *A_out_* is the concentration of drug *A* outside the cell. Thus after the diffusion step, the concentration of *A* inside the cell is


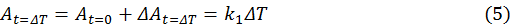


Then a reaction is triggered between *A* and *B*. With the new concentration of *A* inside the cell, the concentration changing rate of *B* can be evaluated by


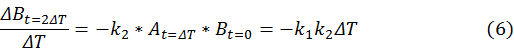


where *k_2_* is the reaction rate constant. The concentration change is the same for *A* during this time period. Thus, after the reaction step the concentration of *A* inside the cell and *B* become


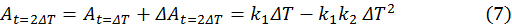


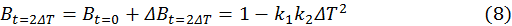


Then start over and calculate concentration change with renewed concentration of *A* and *B* for another loop following this procedure. Typically, at time point *T*, the equations above can be extended as


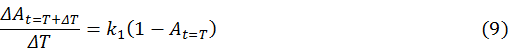


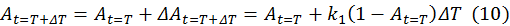


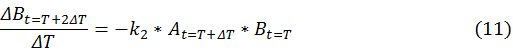


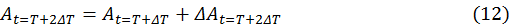


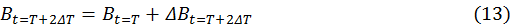


These equations are hard to solve, but they indicate that the concentrations of drug *A* inside the cell and molecular *B* at certain time point are dependent on their concentrations a short time earlier, which depended on the concentrations a short time earlier more. This indication means that we can use mathematical induction to calculate those concentrations at any time point. We have written a MATLAB program based on algorithm described above to achieve this goal.

To evaluate the manner of concentration changes of drug *A* inside the cell and molecular *B* in detail, we fed the model above with some detailed values and obtained the concentration changing curves under different conditions. *ΔT* was set to 0.01, *k_2_* was fixed to 0.5, which was chosen from our experimental observed value, several values (0.01, 0.1, 1 and 10) was selected for *k_1_* to handle its influence. The values were then submitted to the MATLAB program to do the calculations, and curves of concentration against time were obtained.

Besides, when *k_1_* is far larger than *k_2_*, eq. 1 and eq. 2 can be approximately written as


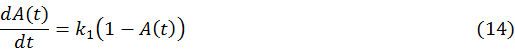


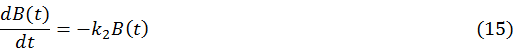


Together with the initiative conditions, we can have


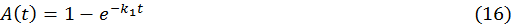


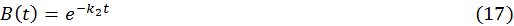


Curves obtained by our model correlate well with the trending curves of eq. 16 and eq. 17 when *k_1_* is larger than *k_2_*.

1. **Supporting references**

1. Panikkanvalappil, S. R. *et al.* Hyperoxia induces intracellular acidification in neonatal mouse lung fibroblasts: Real-time investigation using plasmonically enhanced Raman spectroscopy. *J. Am. Chem. Soc.* **138**, 3779-3788, (2016).

2. Panikkanvalappil, S. R., Hira, S. M., Mahmoud, M. A. & El-Sayed, M. A. Unraveling the biomolecular snapshots of mitosis in healthy and cancer cells using plasmonically-enhanced Raman spectroscopy. *J. Am. Chem. Soc.* **136**, 15961-15968, (2014).

3. Aioub, M. & El-Sayed, M. A. A Real-time surface enhanced Raman spectroscopy study of plasmonic photothermal cell death using targeted gold nanoparticles. *J. Am. Chem. Soc.* **138**, 1258-1264, (2016).

4. Kang, B., Austin, L. A. & El-Sayed, M. A. Observing real-time molecular event dynamics of apoptosis in living cancer cells using nuclear-targeted plasmonically enhanced Raman nanoprobes. *ACS Nano* **8**, 4883-4892, (2014).

5. Kneipp, K., Moskovits, M. & Kneipp, H. *Surface-Enhanced Raman Scattering: Physics and Applications*. (Springer Science & Business Media, 2006).

6. Yuan, H. K. *et al.* Gold nanostars: Surfactant-free synthesis, 3D modelling, and two-photon photoluminescence imaging. *Nanotechnology* **23**, 075102, (2012).

7. Wuelfing, W. P., Gross, S. M., Miles, D. T. & Murray, R. W. Nanometer gold clusters protected by surface-bound monolayers of thiolated poly(ethylene glycol) polymer electrolyte. *J. Am. Chem. Soc.* **120**, 12696-12697, (1998).

8. Busk, M., Pytela, R. & Sheppard, D. Characterization of the integrin alpha v beta 6 as a fibronectin-binding protein. *J. Biol. Chem.* **267**, 5790-5796, (1992).

9. Dehari, H. *et al.* Enhanced antitumor effect of RGD fiber-modified adenovirus for gene therapy of oral cancer. *Cancer Gene Ther.* **10**, 75-85, (2003).

10. Dingwall, C. & Laskey, R. A. Nuclear targeting sequences — a consensus? *Trends Biochem. Sci.* **16**, 478-481, (1991).

11. Robbins, J., Dilworth, S. M., Laskey, R. A. & Dingwall, C. Two interdependent basic domains in nucleoplasmin nuclear targeting sequence: Identification of a class of bipartite nuclear targeting sequence. *Cell* **64**, 615-623, (1991).

12. Kang, B., Afifi, M. M., Austin, L. A. & El-Sayed, M. A. Exploiting the nanoparticle plasmon effect: Observing drug delivery dynamics in single cells via Raman/fluorescence imaging spectroscopy. *ACS Nano* **7**, 7420-7427, (2013).

13. Yu, S.-W. *et al.* Apoptosis-inducing factor mediates poly(ADP-ribose) (PAR) polymer-induced cell death. *Proc. Nat. Acad. Sci. USA* **103**, 18314-18319, (2006).
